# Systematic review, network meta-analysis and economic evaluation of biological therapy for the management of active psoriatic arthritis

**DOI:** 10.1186/1471-2474-15-26

**Published:** 2014-01-20

**Authors:** Matthew Richard Cawson, Stephen Andrew Mitchell, Chris Knight, Henry Wildey, Dean Spurden, Alex Bird, Michelle Elaine Orme

**Affiliations:** 1 RTI Health Solutions, Sheffield, UK; 2 Abacus International, 6 Talisman Business Centre, Talisman Road, Bicester OX26 6HR, UK; 3 Pfizer Ltd, Surrey, UK; 4 ICERA Consulting Ltd, Swindon, UK

## Abstract

**Background:**

An updated economic evaluation was conducted to compare the cost-effectiveness of the four tumour necrosis factor (TNF)-α inhibitors adalimumab, etanercept, golimumab and infliximab in active, progressive psoriatic arthritis (PsA) where response to standard treatment has been inadequate.

**Methods:**

A systematic review was conducted to identify relevant, recently published studies and the new trial data were synthesised, via a Bayesian network meta-analysis (NMA), to estimate the relative efficacy of the TNF-α inhibitors in terms of Psoriatic Arthritis Response Criteria (PsARC) response, Health Assessment Questionnaire (HAQ) scores and Psoriasis Area and Severity Index (PASI). A previously developed economic model was updated with the new meta-analysis results and current cost data. The model was adapted to delineate patients by PASI 50%, 75% and 90% response rates to differentiate between psoriasis outcomes.

**Results:**

All four licensed TNF-α inhibitors were significantly more effective than placebo in achieving PsARC response in patients with active PsA. Adalimumab, etanercept and infliximab were significantly more effective than placebo in improving HAQ scores in patients who had achieved a PsARC response and in improving HAQ scores in PsARC non-responders. In an analysis using 1,000 model simulations, on average etanercept was the most cost-effective treatment and, at the National Institute for Health and Care Excellence willingness-to-pay threshold of between £20,000 to £30,000, etanercept is the preferred option.

**Conclusions:**

The economic analysis agrees with the conclusions from the previous models, in that biologics are shown to be cost-effective for treating patients with active PsA compared with the conventional management strategy. In particular, etanercept is cost-effective compared with the other biologic treatments.

## Background

Psoriatic arthritis (PsA) is a chronic systemic inflammatory disease characterised by joint involvement and several heterogeneous extra-articular manifestations, including enthesitis, dactylitis and dermatological involvement of the skin and nails ([1]). The broad involvement of articular and non-articular sites can have a significant impact on patients’ function and quality of life [2]. The presentation of PsA has been categorised into five overlapping clinical patterns; oligoarthritis (22% to 37% of patients); polyarthritis (36% to 41% of patients); arthritis of distal interphalangeal joints (up to 20% of patients); spondylitis (7% to 23% of patients); and arthritis mutilans (approximately 4%) [3,4]. The prevalence of PsA is greater among psoriasis patients, with a prevalence rate spanning a wide range from 7% to 26% [5]. Around seventy per cent of PsA patients develop joint complications usually around ten years after developing skin symptoms, whereas, 10-15% of patients suffer from joint damage before developing psoriasis, and in the remaining 10-15% of patients, these symptoms may manifest simultaneously [6].

There are a number of published recommendations for the management of PsA [7,8]. Treatment is dependent on the type and severity of the skin and joint involvement. Patients with mild-to-moderate PsA are frequently given non-steroidal anti-inflammatory drugs (NSAIDs) and intra-articular steroid injections. Patients with more severe PsA and persistent arthritis not responding to NSAIDs are treated with disease-modifying anti-rheumatic drug (DMARD) therapy. Methotrexate, sulphasalazine and cyclosporine-A are the commonly used DMARDs [9].

More recently, newer treatments targeting the inflammatory cascade and preventing disease progression have been introduced including tumour necrosis factor (TNF)-α inhibitors. These drugs are used as monotherapy or in combination with the traditional nonbiologic DMARDs such as methotrexate. The combination regimen is used in patients with severe disease or with ongoing joint damage and disease progression [6]. While there is evidence to suggest that treatment with concomitant methotrexate is beneficial compared with TNF-α monotherapy (resulting from fewer withdrawals due to adverse events) [10], this has not been a universal finding [11].

There are currently no head-to-head randomised controlled trials (RCTs) comparing the TNF-α inhibitors to each other and therefore attempts to compare the relative efficacy and safety of these agents have relied upon a qualitative review of the published evidence or meta-analytic techniques [12]. A recently published meta-analysis assessing the relative efficacy of the currently available TNF-α inhibitors concluded that etanercept was the most efficacious treatment (as measured by American College of Rheumatology (ACR) response) compared with infliximab and adalimumab [13]. RCT data are also available for the TNF-α inhibitor golimumab [14] which has recently been recommended by the National Institute for Health and Care Excellence (NICE) as an option for the treatment of active and progressive PsA in adults, in the UK [15]. An economic evaluation developed for the NICE review concluded that etanercept, infliximab and adalimumab are cost-effective versus palliative care [16,17]. However, it is unclear how cost-effective golimumab is compared with palliative care (conventional management strategy) and head-to-head with these three biologics.

Therefore, this paper presents a new economic evaluation supported by an updated systematic review and meta-analysis that included recent data for golimumab, the objective being to determine the relative cost-effectiveness of all UK licensed biological disease-modifying anti-rheumatic drugs (bDMARDs) for the treatment of active, progressive PsA in patients with inadequate response to previous DMARDs. The paper presents the results from the updated meta-analysis for all clinical measures of efficacy used in the economic model (note that other clinical measures such as ACR response are reported elsewhere [18]). The meta-analysis results were used in a revised economic model which updates the previous NICE models [16,17] to provide a cost-effectiveness comparison of all four TNF-α inhibitors adalimumab, etanercept, golimumab and infliximab.

## Methods

A comprehensive systematic review was conducted to identify RCTs of bDMARDs for the treatment of people with active PsA (defined globally as one or more tender and inflamed joint and/or tender enthesis point and/or dactylitic digit and/or inflammatory back pain [8]) who have responded inadequately to previous DMARDs.

Structured literature searches were conducted for the following databases (accessed October 31^st^ 2011): The Cochrane Library (including Cochrane Reviews, the Database of Abstracts of Reviews of Effects (DARE), the Cochrane Central Register of Controlled Trials (CENTRAL), the Health Technology Assessment Database (HTA)), Ovid MEDLINE^(R)^ In-Process & Other Non-Indexed Citations and Ovid MEDLINE^(R)^ (1948 to present), and OVID EMBASE (1980 to present). Search terms included those for the disease (‘psoria* adj arthrit*’), interventions (‘DMARD’ or etanercept or infliximab or adalimumab or golimumab) and study type (randomi?ed controlled study). In addition the following conference proceedings were hand-searched (2008–2011 inclusive): World Psoriasis and Psoriatic Arthritis Conference, ACR, European League against Rheumatism (EULAR), British Society for Rheumatology (BSR). Reference lists of included studies and previously published systematic reviews were also examined for relevant citations. Studies (full publications or conference abstracts in the absence of a full publication) were included in the systematic review if they met the pre-defined inclusion criteria (see Additional file 1).

Potentially relevant studies (based on abstract/title) were examined in full by two independent reviewers. Relevant outcome data were extracted by two independent reviewers and any disputes resolved by consensus.

The quality of the included RCTs was assessed according to the methodology checklist detailed in the NICE Guidelines Manual 2009 [19,20]. In brief, this assesses the likelihood of selection, attrition, detection, and performance bias.

The methodology for the meta-analysis was as per Rodgers et al., 2011 [16], and the recommended methods published by the NICE Decision Support Unit [21]. The following outcomes were evaluated to assess the relative efficacy of the bDMARDs: Psoriatic Arthritis Response Criteria (PsARC) response; change in Health Assessment Questionnaire (HAQ) score from baseline (change in HAQ score for all patients; HAQ score conditional on PsARC response); Psoriasis Area and Severity Index (PASI).

The analysis of the PsARC and PASI dichotomous (patient count) outcomes was conducted on an intent-to-treat basis. For the continuous HAQ data, the mean change and standard error in mean change were used. An ordered multinomial analysis of PASI score at follow-up was conducted based on the number of patients in four PASI response categories: PASI score improvement of 0% to 50%, PASI score improvement of 50% to 75%, PASI score improvement of 75% to 90%, and PASI score improvement of 90% to 100%. An ordered multinomial model makes more efficient use of categorical data than a binomial analysis of each category separately. Note that in the clinical trials, PASI scores at follow-up were reported for patients who had ≥3% body surface area (BSA) involvement at baseline or a PASI score ≥2.5 at baseline.

Study inclusion criteria allowed the inclusion of outcomes measured after at least 12 weeks of follow-up, since clinical response should have occurred by that point, and outcomes reported for up to 24 weeks of follow-up. Data from the 12–16 week follow-up was used in the basecase as outcomes at this time point are reported for most studies (and in some studies this was the pre-crossover follow-up point). In studies with an early escape design, the 24 week data were included provided the pre-crossover observation was carried forward.

A Bayesian network meta-analysis (NMA) using uninformative priors was conducted in WinBUGS version 1.4.1. [22-24]. Whereas standard meta-analyses evaluate the relative efficacy of just two treatments based on head-to-head trials only, NMA comprises an extension of these methods in which treatment effects are calculated for a network of treatments [25-27]. Hence NMAs estimate the relative efficacy of each treatment in the network compared with all other treatments. An NMA builds on the principles of indirect comparisons whilst preserving trial randomisation [28-30].

For the analysis of HAQ conditional on PsARC response, it was assumed that TNF-α inhibitors have different treatment effects conditional on PsARC response. For the analysis of ordered PASI response thresholds, a number of assumptions were made to facilitate modelling: a common-effects model was used to estimate the baseline response estimated using data from placebo non-responders (i.e. those receiving placebo and not achieving PASI 50); common effects were assumed for each treatment (etanercept, golimumab, infliximab and adalimumab); thresholds were assumed to be fixed across trials and the baseline latent variable was assumed fixed.

For full details on the methodology the reader is referred to Rodgers et al., 2011 [16].

The economic model is a Markov cohort model using 3-month cycles, and is based on a previously published model structure [16,17]. The original model was used to evaluate the cost-effectiveness of etanercept, adalimumab, infliximab and palliative care (conventional management strategy). This structure was adapted to include additional PASI response (PASI 50 and PASI 90) levels to provide more sensitivity to the psoriasis element of the disease and the cost and efficacy data were updated to include the recent evidence for golimumab. Model methods and assumptions are consistent with the original model [16,17]. For biologic treatment, the PsARC response criterion is used to determine a response in the first three months. Following this initial assessment the impact on the two elements of PsA, arthritis and psoriasis, are assessed using the HAQ and PASI respectively. The change in HAQ and PASI scores are assumed to be relative to their baseline values (assumed to be 1.05 and 7.5, respectively as per Bojke et al., 2011 [17]). Patients on palliative care are assumed to follow the natural history of HAQ and PASI [16,17].

**Figure 1:**
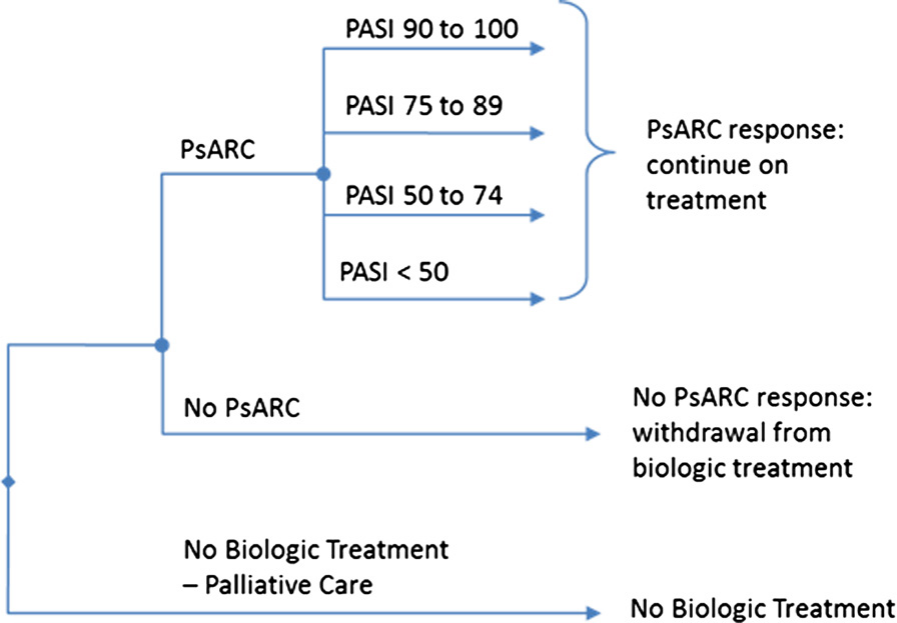
Markov model structure.

The model structure as shown in Figure 1 follows a cohort of patients with PsA as follows:

• Initial 3 month period: the probability of a PsARC response is used to determine the proportion of patients who enter the subsequent model pathways.

• If PsARC response is achieved, patients remain on treatment and it is assumed the gain in HAQ is maintained while biologic therapy is maintained [16,17].

• If PsARC response is not achieved, patients revert to the conventional management strategy and it is assumed that the rebound is equal to gain, after which natural HAQ progression is assumed.

• Subsequent follow-up: modelled as three-month cycles (up to 159 cycles = 40 years total follow-up).

• During each subsequent period there is a probability of withdrawal from therapy which is assumed to be the same for all treatments [16,17].

• Quality-adjusted life years (QALYs) for each treatment are derived by mapping the estimated HAQ and PASI scores onto utilities using the same equation as for the other models (Expected Utility = 0.897-0.298 x HAQ -0.004 x PASI [(SE) (0.006) (0.006) (0.0003)]) [16,17].

The model structure and assumptions differ from the Bojke et al., 2011 model [17] in a number of elements. The most significant regards the psoriasis element of the model. Where the previously published model [17] uses PASI 75 only, the new model delineates PASI further by using PASI 50, 75 and 90 response rates derived from the meta-analysis. This makes the model more sensitive to differences in psoriasis outcomes. Patients continue on biologic treatment even if they do not meet a given PASI response level. Patients will remain on treatment for as long as a PsARC response is sustained and receive a proportionate gain in PASI from baseline over the duration of this period (see Rodgers et al., 2011 [16] for further detail).

Rodgers et al., 2011 indicated that PsARC and PASI response may not be independent [16]. Hence the relationship between PsARC and PASI was taken from the Rodgers et al., 2011 HTA publication [16] as the assumptions regarding the PsARC and PASI relationship were not explicit in the Bojke et al., 2011 paper [17]. Furthermore the proportion of males with PsA entering the model was also unclear and thus we assumed a 50:50 split. This assumption only affects the all-cause general mortality calculation which was applied to all treatments and was estimated from UK life tables [31].

All drug [32] and attendance costs were updated to 2012, while resource costs and direct costs were uplifted to 2011 using the Hospital and Community Health Services Index (HCHS) [33] and the annual discount rate is 3.5% for both costs and QALYs [20]. Dosage was consistent with current guidance (BNF, 64): infliximab (in combination with methotrexate) dosage is 5 mg/kg and the model assumes an average weight of 75 kg. The updated cost data and other data in the model are shown in Table 1. A Monte Carlo simulation methodology was used for a probabilistic sensitivity analysis. All parameters in the model were characterised by probabilistic distributions as described in Bojke et al., 2011 [17] (see Table 1).

**Table 1:** Economic model drug cost data and other economic model data

**Drug cost, £**^**+**^	**ETN**	**INF**	**ADA**	**GOL**	**PSA**	**Ref**
First 3 months	2323.75	5043.21	2288.91	2288.91	Normal	BSR guidelines [34] and MIMS [35]
Months 4-6	2323.75	2693.34	2288.91	2288.91		
Subsequent 3 months	2323.75	2693.34	2288.91	2288.91		
**Other data**			**Mean**	**SE**	**PSA**	**Ref**
Change in cost for 1 U change in HAQ			106.5	69.3	Normal	Kobelt 2002 [36]
Three-month cost for mild-to-moderate psoriasis if uncontrolled by biologics			205.2	9.3	Normal	DoH 2007/2008 [37]
Three-month cost for psoriasis in remission			16.5	1	Normal	Hartman 2003 [38]
Change in HAQ while on treatment per 3-month period			0	0.02	Normal	Bojke 2011 [17] (expert elicitation)
Change in HAQ while not on treatment per 3-month period			0.018	0.007	Gamma	Bojke 2011 [17] (NOAR estimate)
Log withdrawal rate from biologics per year			-1.823	0.2044	Normal	Bojke 2011 [17] (Registers)
Probability of PsARC response on placebo			0.249	0.0384	Beta	Bojke 2011 [17] (Evidence synthesis)
Change in HAQ given a PsARC response on placebo			-0.2436	0.04746	Normal	Bojke 2011 [17] (Evidence synthesis)
Probability of PASI 75 response on placebo			0.044	0.009	Beta	Bojke 2011 [17] (Evidence synthesis)

^+^All costs inflated to 2010/11 values (£’s); ADA, Adalimumab 40 mg/2 weeks; BSR, British Society of Rheumatology; ETN, Etanercept 2×25 mg/week; INF, Infliximab 5 mg/kg/8 weeks; GOL, Golimumab 50 kg/4 weeks; HAQ, health assessment questionnaire; PsARC, Psoriatic Arthritis Response Criteria; PASI, Psoriasis Area and Severity Index; PSA, distribution used in probabilistic sensitivity analysis.

## Results and discussion

A total of 2,099 potentially relevant citations were identified for inclusion in the systematic review on the basis of the database search, of which 2,036 were excluded on the basis of title or abstract (see Additional file 2). On re-application of the review inclusion criteria to the 63 full-text papers, a further 38 were excluded. Five additional publications were identified as a result of searching conference proceedings and the grey literature. Therefore 30 publications detailing 12 RCTs [14,39-49] met the inclusion criteria and were included in the systematic review.

On completion of the data extraction, a feasibility assessment was conducted to assess a priori which studies were sufficiently homogenous to be combined in a robust meta-analysis. On review of the 12 studies with regard to study design, inclusion criteria, treatment regimens and reported outcomes, five studies [41,42,45,46,49] were excluded for either having a short follow-up time [49], not having a placebo arm required for inclusion in the network analysis [41,42], or inclusion criteria [45,46], leaving seven studies [14,39,40,43,44,47,48] in the potential evidence network.

• Two studies examining adalimumab 40 mg every other week (n = 204) versus placebo (n = 211) [47,48]

• Two studies examining etanercept 25 mg twice weekly (n = 131) versus placebo (n = 134) [39,40]

• A single study examining golimumab 50 mg (n = 146) every 4 weeks versus placebo (n = 113) plus a third unlicensed arm (golimumab 100 mg every 4 weeks: n = 146) [14]

• Two studies examining infliximab 5 mg/kg (n = 152) versus placebo (n = 152) [43,44]

The degree of clinical heterogeneity between the seven included trials in terms of joint and skin disease severity and functional status was reasonable and therefore the degree of exchangeability between the trials for the purposes of the meta-analysis was good. The seven studies were generally of good quality: randomisation, blinding, concealment of allocation and intention-to-treat analyses were adequate in most trials. In order to conduct the meta-analysis for the HAQ outcome, additional data were also extracted from two secondary publications (Rodgers et al., 2011 [16] and Cummins et al., 2011 [50]). See the online appendix for a summary of the studies (Additional file 3) and the data extracted for the meta-analysis (Additional files 4 and 5).

Table 2 summarises the NMA results which were used in the economic evaluation alongside a comparison with the results from the NMA by Rodgers et al., 2011 [16]. (Note that results for the ACR outcomes which are not used in the economic model are reported in Spurden et al., 2012 [18]).

**Table 2:** **Results of fixed-effect network meta-analysis and comparison with previous network meta-analysis by Rodgers et al., 2011**[16]

		**Placebo**	**Adalimumab 40 mg/2 weeks**	**Infliximab 5 mg/kg/8 weeks**	**Golimumab 50 kg/4 weeks**	**Etanercept 2x25 mg/week**
PsARC response:	Odds ratio versus placebo OR (95% CrI)	NA	4.28 (2.83, 6.57)^†^	9.97 (5.95, 17.08)^†^	10.33 (5.84, 19.04)^†^	7.74 (4.5, 13.67)^†^
	Probability (95% CrI)^+^	0.26 (0.22, 0.29)	0.59 (0.48, 0.70)	0.77 (0.66, 0.86)	0.78 (0.66, 0.87)	0.73 (0.60, 0.83)
	Comparison with Rodgers 2011 [12]	0.25 (0.18, 0.32)	0.59 (0.44, 0.71)	0.80 (0.67, 0.89)	NA	0.71 (0.57, 0.83)
Change in HAQ conditional on PsARC response	WMD versus placebo non-responders | PsARC responders (95% CrI)*	-0.26 (-0.32, -0.21)^†^	-0.49 (-0.58, -0.40)^†^	-0.66 (-0.77, -0.55)^†^	-0.44 (-0.59, -0.29)^†^	-0.64 (-0.77, -0.51)^†^
	Comparison with Rodgers 2011 [12]	-0.24 (-0.34, -0.15)^†^	-0.48 (-0.60, -0.35)^†^	-0.66 (-0.79, -0.52)^†^	NA	-0.63 (-0.81, -0.46)^†^
	WMD versus placebo non-responders | PsARC non-responders (95% CrI)*	NA	-0.14 (-0.24, -0.03)^†^	-0.20 (-0.31, -0.08)^†^	-0.06 (-0.18, 0.06)	-0.20 (-0.35, -0.050)^†^
	Comparison with Rodgers 2011 [12]	NA	-0.13 (-0.26, -0.00)^†^	-0.19 (-0.33, -0.06)^†^	NA	-0.19 (-0.381, 0.00)
PASI50	Probability (95% CrI)^+^	0.12 (0.09, 0.16)	0.71 (0.51, 0.86)	0.90 (0.80, 0.96)	0.71 (0.50, 0.87)	0.40 (0.16, 0.73)
	Comparison with Rodgers 2011 [12]	0.13 (0.09,0.18)	0.74 (0.55,0.88)	0.91 (0.82,0.97)	NA	0.40 (0.24,0.59)
PASI75	Probability (95% CrI)^+^	0.05 (0.03, 0.07)	0.47 (0.27, 0.68)	0.77 (0.59, 0.89)	0.46 (0.26, 0.70)	0.19 (0.06, 0.49)
	Comparison with Rodgers 2011 [12]	0.04 (0.03,0.07)	0.48 (0.28,0.69)	0.77 (0.59,0.90)	NA	0.18 (0.09,0.31)
PASI90	Probability (95% CrI)^+^	0.02 (0.01, 0.03)	0.24 (0.12, 0.44)	0.54 (0.34, 0.75)	0.23 (0.11, 0.46)	0.08 (0.02, 0.26)
	Comparison with Rodgers 2011 [12]	0.02 (0.01,0.03)	0.26 (0.12,0.45)	0.56 (0.35,0.77)	NA	0.07 (0.03,0.15)

CrI, credible interval (Bayesian probability interval); HAQ, health assessment questionnaire; NMA, network meta-analysis; OR, odds ratio; PsARC, Psoriatic Arthritis Response Criteria; PASI, Psoriasis Area and Severity Index; WMD, weighted mean differences; NA, not applicable ^†^significant result based on 95% CrI. ^+^Average results were used in the economic model; the probabilistic sensitivity analysis used a beta distribution. *Average results were used in the economic model; the probabilistic sensitivity analysis used a normal distribution. Results for direct, fixed-effect meta analysis for PASI 70/75/90 are reported in Additional files 6, 7 and 8.

Similar results were obtained in the sensitivity analysis using data up to week 24 (not shown). All four licensed TNF-α inhibitors were significantly more effective than placebo in achieving PsARC response in patients with active PsA (Table 2, Additional file 9). Etanercept and infliximab were significantly more effective than placebo in improving HAQ scores (Additional file 10). Golimumab was not significantly better than placebo in improving HAQ scores [18].

For the change in HAQ conditional on PsARC response, adalimumab, etanercept and infliximab were significantly more effective than placebo in improving HAQ scores in patients who had achieved a PsARC response but also in improving HAQ scores in PsARC non-responders (Table 2. Additional file 11). Golimumab was not significantly better than placebo in improving HAQ scores in patients who had achieved a PsARC response (the 95% CrI for golimumab PsARC responders overlaps the 95% CrI for placebo PsARC responders, see Table 2).

The meta-analysis for conditional change in HAQ based on PsARC response produced similar results to the meta-analysis reported in Rodgers et al., 2011 [16] which assessed three of the TNF-α inhibitors. The Rodgers NMA did not report the relative treatment effects for the PsARC analysis but instead reported the absolute probability of PsARC, which requires an estimate of the underlying background response (placebo response) as well as the treatment effects relative to placebo. Table 2 shows a comparison of the absolute probability of PsARC reported in Rodgers et al., 2011 versus the corresponding results from our NMA. The PsARC analysis results were sensitive to the choice of infliximab data since the placebo response at week 14 in the IMPACT study [44] was lower than the 16 week data reported in the publication (analysis not shown). This affected both the underlying background response and the relative effect of infliximab. However, this did not alter the overall ranking of the TNF-α inhibitors.

An incremental analysis was conducted where treatments are listed in order of clinical efficacy and the incremental cost-effectiveness ratio (ICER = ratio of difference in costs and difference in QALYs) of each intervention is calculated by comparing it to the next most effective intervention (see Table 3). Based on this incremental analysis golimumab was dominated by etanercept (etanercept costs less and is more effective than golimumab); adalimumab was extendedly dominated by etanercept (ICER is greater than that of the more effective intervention); etanercept was cost-effective (based on an ICER < £20,000- £30,000) compared with the conventional management strategy and infliximab was not cost-effective compared with etanercept. These findings are similar to those reached by Bojke et al., 2011 [17].

**Table 3:** Results of incremental economic analysis

**Treatment**	**Mean Cost**	**Mean QALYs**	**Inc. Cost**	**Inc. QALY**	**ICER**	**ICER v Conventional**
At 10 years						
Conventional management strategy	£15,587	3.8	-	-	-	-
Adalimumab	£39,070	4.5	£23,484	0.7	Ext Dom’d	£31,830
Golimumab	£45,990	4.7	£6,920	0.2	Dom’d	£33,178
Etanercept	£44,701	4.8	£29,115	1.0	£28,917	£28,917
Infliximab	£56,009	4.9	£11,308	0.1	£86,499	£35,534
At 40 years						
Conventional management strategy	£43,391	5.2	-	-	-	-
Adalimumab	£69,332	6.7	£25,941	1.5	Ext Dom’d	£17,222
Golimumab	£76,976	7.1	£7,629	0.4	Dom’d	£17,435
Etanercept	£75,563	7.2	£32,171	2.0	£16,426^a^	£16,426
Infliximab	£88,362	7.4	£12,799	0.2	£62,527^b^	£20,789

Dom’d, dominated (Treatment costs more and is less effective than the other intervention); Ext Dom’d, extendedly dominated (ICER is greater than that of the more effective intervention); ICER, incremental cost-effectiveness ratio; QALY, quality-adjusted life years.

^a^Compared with the next most effective strategy (excluding the extendedly dominated and dominated options) i.e. conventional management strategy. ^b^Compared with the next most effective strategy (excluding the extendedly dominated and dominated options) i.e. etanercept.

**Figure 2:**
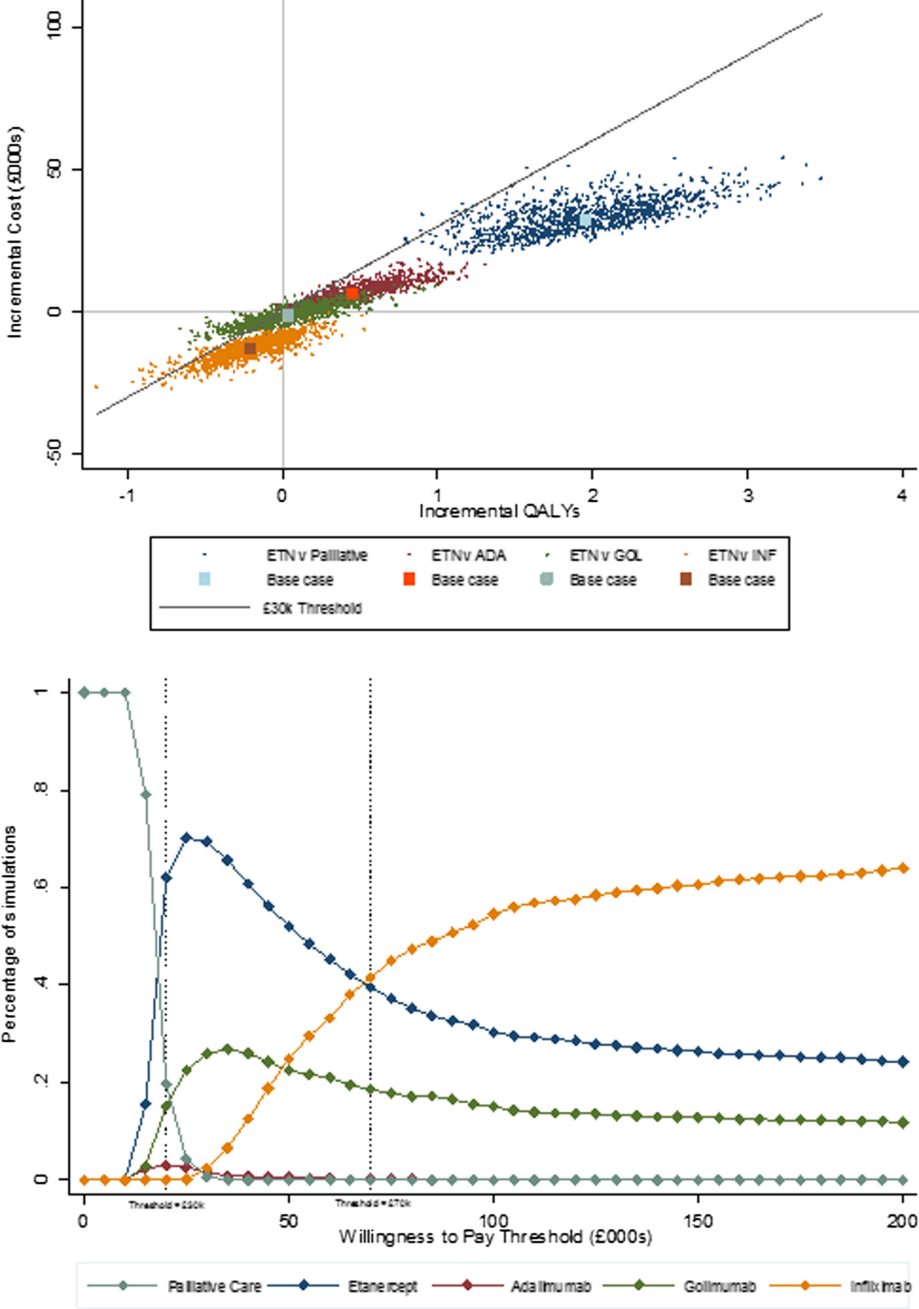
**Results from 1,000 model simulations.** Upper panel: incremental cost and QALYs for etanercept plotted on cost-effectiveness plane; lower panel: percentage of simulations where treatment has highest net monetary benefit at varying WTP thresholds.

Probabilistic sensitivity analysis was undertaken to evaluate the robustness of the deterministic results and compared all the treatments. Figure 2, upper panel shows the incremental cost and QALYs of etanercept versus other treatments and Figure 2, lower panel shows the proportion of simulations with the highest net monetary benefit (NMB) based on willingness-to-pay (WTP, NMB = (QALYs*WTP – cost) at different WTP thresholds. In an analysis using 1,000 model simulations (40 year model), on average, etanercept was the most cost-effective treatment and, at a WTP threshold of between £20,000 and £70,000, etanercept is the preferred option. When considering the NICE WTP threshold of between £20,000 and £30,000 per QALY gained, the probability that etanercept was the preferred option was between 62% and 70% [19].

A limitation of any meta-analysis is the underlying assumption that trials and outcomes are sufficiently similar to allow data to be pooled. This applies to any method of data synthesis, not just NMA. There is a relative paucity of data for both conventional and biological DMARDs [51] which limits the number of studies available for the meta-analysis. This reduces the capacity for a meta-analysis to estimate study heterogeneity. As there were few studies that qualified for inclusion, common-effects models were used in the meta-analysis in Rodgers et al., 2011 [16] since these models provided a good fit based on the deviation information criterion (DIC) output from WinBUGs, as well as good convergence without autocorrelation issues. In this analysis, with the addition of one extra study, the models were found to have good fit based on DIC, but also based on average posterior mean residual deviance.

The lack of data meant we were unable to conduct a formal investigation of potential sources of study heterogeneity. The treatment effect estimates may be affected by potentially non-comparable populations, e.g. different severity of disease, different patterns of disease, different duration of disease, or by the use of different background treatments, as well as differences in study design and in the objective measurement of efficacy outcomes. In particular the Outcome Measures in Rheumatology (OMERACT) core set of domains are not consistently reported in all RCTs reporting on treatments for PsA [51,52]. Although development of a validated, disease-specific composite measure incorporating appropriate disease domains into a single measure remains challenging [53], novel composite measures have recently been proposed to assess disease activity in patients with PsA [54]. Trials should incorporate objective, measurable and relevant outcomes though it remains to be determined which outcomes are of most importance to patients.

The model outlined here is a replication of previous models that has included the biologic golimumab, in addition to etanercept, infliximab and adalimumab. However, the model has expanded the impact of the psoriasis element of the disease by including PASI 50 and PASI 90 as well as PASI 75 scores which affect the estimation of quality of life utility scores via the mapping algorithm. The results of this model show that under base-case assumptions, etanercept would be considered the most cost-effective strategy for patients with PsA and minimal, mild-to-moderate or moderate-to-severe skin involvement. This result is fairly robust as the probability of etanercept being cost-effective at a WTP threshold of £30,000 per QALY is 70%. This result is similar to those produced in the early model versions that excluded golimumab [16,17] and confirms the results from a real-world study where TNF-α inhibitors were reported to be cost-effective in the treatment of PsA patients inadequately managed with conventional agents [55].

## Conclusions

In these selected studies, all bDMARDs were significantly more effective than placebo in achieving PsARC response in patients with active PsA. Across the studies included in the analysis, etanercept and infliximab were significantly more effective than placebo in improving HAQ scores in all patients regardless of PsARC response and in subgroups who achieved a PsARC response and PsARC non-responders. The probabilistic analysis from the model showed that, with a £30,000 per QALY WTP threshold, etanercept is a cost-effective treatment for patients with active PsA compared with the other biologic treatments of infliximab, adalimumab and golimumab and also compared with the conventional management strategy.

## Competing interests

This study was sponsored by Pfizer Ltd, UK. Matthew Cawson, Henry Wildey, Chris Knight, Michelle Orme and Stephen Mitchell were paid consultants to Pfizer Ltd, UK. Dean Spurden and Alex Bird are paid employees of Pfizer Ltd.

## Authors’ contributions

MO conducted the meta-analysis and assisted in drafting the manuscript. SM conducted the systematic review which provided data for the meta-analysis and assisted in drafting the manuscript. MC, HW, and CK developed the economic model and assisted in drafting the manuscript. DS and AB assisted in drafting and revising the manuscript. All authors read and approved the final manuscript.

## Pre-publication history

The pre-publication history for this paper can be accessed here:

http://www.biomedcentral.com/1471-2474/15/26/prepub

## Supplementary Material

Additional file 1: Table S4 Selection criteria for studies included in the meta-analysis. Click here for file

Additional file 2: Figure S3 Flow chart of inclusions/exclusions (RCTs). Click here for file

Additional file 3: Table S5 Summary of studies included in the meta-analysis. Click here for file

Additional file 4: Table S6 Data used in NMA: PsARC response, change in HAQ compared to baseline and PASI 50/75/90. Click here for file

Additional file 5: Table S7 Data used in NMA: Conditional Data for change in HAQ | PsARC response compared to baseline. Click here for file

Additional file 6: Figure S6 Forest plot of direct fixed-effect meta-analysis: Odds ratio of PASI50 compared to placebo (follow-up at 12–16 weeks). Click here for file

Additional file 7: Figure S7 Forest plot of direct fixed-effect meta-analysis: Odds ratio of PASI75 compared to placebo (follow-up at 12–16 weeks). Click here for file

Additional file 8: Figure S8 Forest plot of direct fixed-effect meta-analysis: Odds ratio of PASI90 compared to placebo (follow-up at 12–16 weeks). Click here for file

Additional file 9: Figure S4 Forest plot of direct fixed-effect meta-analysis: Odds ratio of PsARC response compared to placebo (follow-up at 12–16 weeks). Click here for file

Additional file 10: Figure S5 Forest plot of direct fixed-effect meta-analysis: Difference in HAQ compared to placebo (follow-up at 12–16 weeks). Click here for file

Additional file 11: Figure S9 Forest plot of direct fixed-effect meta-analysis: Difference in HAQ compared to placebo stratified by PsARC response (follow-up at 12–16 weeks). Click here for file

## References

[B1] Day MS Nam D Goodman S Su EP Figgie M Psoriatic arthritis J Am Acad Orthop Surg 2012 20 1 28 37 10.5435/JAAOS-20-01-028 22207516

[B2] Rosen CF Mussani F Chandran V Eder L Thavaneswaran A Gladman DD Patients with psoriatic arthritis have worse quality of life than those with psoriasis alone Rheumatology (Oxford) 2012 51 3 571 6 10.1093/rheumatology/ker365 22157469

[B3] Gladman DD Shuckett R Russell ML Thorne JC Schachter RK Psoriatic arthritis (PSA)–an analysis of 220 patients Q J Med 1987 62 238 127 41 3659255

[B4] Torre Alonso JC Rodriguez Perez A Arribas Castrillo JM Ballina Garcia J Riestra Noriega JL Lopez LC Psoriatic arthritis (PA): a clinical, immunological and radiological study of 180 patients Br J Rheumatol 1991 30 4 245 50 10.1093/rheumatology/30.4.245 1863819

[B5] Prey S Paul C Bronsard V Puzenat E Gourraud PA Aractingi S Assessment of risk of psoriatic arthritis in patients with plaque psoriasis: a systematic review of the literature J Eur Acad Dermatol Venereol 2010 24 Suppl 2 31 5 20443998 10.1111/j.1468-3083.2009.03565.x

[B6] Young MS Furfaro N Rai A Diagnosis and Management of Psoriatic Arthritis A Practical Review Journal of the Dermatology Nurses' Association 2009 1 5 283 293 2009 September/October 10.1097/JDN.0b013e3181ba2dba

[B7] Ritchlin CT Kavanaugh A Gladman DD Mease PJ Helliwell P Boehncke WH Treatment recommendations for psoriatic arthritis Ann Rheum Dis 2009 68 9 1387 1394 2009 Sep 10.1136/ard.2008.094946 18952643 PMC2719080

[B8] Gossec L Smolen JS Gaujoux-Viala C Ash Z Marzo-Ortega H van der Heijde D European League Against Rheumatism recommendations for the management of psoriatic arthritis with pharmacological therapies Ann Rheum Dis 2012 71 1 4 12 10.1136/annrheumdis-2011-200350 21953336

[B9] Miedany YE Recent Developments in Management of Psoriatic Arthritis Curr Rheumatol Rev 2005 1 1 9 19 10.2174/1573397052954154

[B10] Kristensen LE Gulfe A Saxne T Geborek P Efficacy and tolerability of anti-tumour necrosis factor therapy in psoriatic arthritis patients: results from the South Swedish Arthritis Treatment Group register Ann Rheum Dis 2008 67 3 364 9 17644547 10.1136/ard.2007.073544

[B11] Spadaro A Ceccarelli F Scrivo R Valesini G Life-table analysis of etanercept with or without methotrexate in patients with psoriatic arthritis Ann Rheum Dis 2008 67 11 1650 1 10.1136/ard.2007.085951 18854519

[B12] Saad AA Symmons DP Noyce PR Ashcroft DM Risks and benefits of tumor necrosis factor-alpha inhibitors in the management of psoriatic arthritis: systematic review and metaanalysis of randomized controlled trials J Rheumatol 2008 35 5 883 90 18381787

[B13] Migliore A Bizzi E Broccoli S Lagana B Indirect comparison of etanercept, infliximab, and adalumimab for psoriatic arthritis: mixed treatment comparison using placebo as common comparator Clin Rheumatol 2012 31 1 193 4 10.1007/s10067-011-1862-7 22005889

[B14] Kavanaugh A McInnes I Mease P Krueger GG Gladman D Gomez-Reino J Golimumab, a new human tumor necrosis factor alpha antibody, administered every four weeks as a subcutaneous injection in psoriatic arthritis: Twenty-four-week efficacy and safety results of a randomized, placebo-controlled study Arthritis Rheum 2009 60 4 976 86 10.1002/art.24403 19333944

[B15] National Institute for Health and Clinical Excellence Golimumab for the treatment of psoriatic arthritis 2011 London: NICE Available from: http://www.nice.org.uk/nicemedia/live/13441/54169/54169.pdf

[B16] Rodgers M Epstein D Bojke L Yang H Craig D Fonseca T Etanercept, infliximab and adalimumab for the treatment of psoriatic arthritis: a systematic review and economic evaluation Health Technol Assess 2011 15 10 1 329 February 2011 21333232 10.3310/hta15100 PMC4781419

[B17] Bojke L Epstein D Craig D Rodgers M Woolacott N Yang H Modelling the cost-effectiveness of biologic treatments for psoriatic arthritis Rheumatology (Oxford) 2011 50 Suppl 4 iv39 iv47 21859705 10.1093/rheumatology/ker245

[B18] Spurden D Orme ME Mitchell S Bird A Systematic Review and network meta-analysis of biological therapy for the management of active psoriatic arthritis [SAT0298] Ann Rheum Dis 2012 71 3 573

[B19] NICE. NICE Guidelines Manual, Appendix D http://www.nice.org.uk/media/633/21/The_guidelines_manual_2009_-_Appendix_D_Methodology_checklist_-_randomised_controlled_trials.pdf. 2009

[B20] National Institute for Health and Clinical Excellence (NICE) Updated guide to the methods and of technology appraisal 2008 London: NICE 27905712

[B21] Dias S Welton NJ Sutton AJ Ades AE NICE DSU Technical Support Document 2: A Generalised Linear Modelling Framework for Pairwise and Network Meta-analysis of Randomised Controlled Trials 2011 27466657

[B22] Sutton AJ Abrams KR Bayesian methods in meta-analysis and evidence synthesis Stat Methods Med Res 2001 10 4 277 303 10.1191/096228001678227794 11491414

[B23] Spiegelhalter DJ Abrams KR Myles JP Bayesian Approaches to Clinical Trials and Health Care Evaluation 2004 Chichester, UK: Wiley

[B24] Bayesian inference using Gibbs Sampling (BUGS) WinBUGS with DoodleBUGS version 1.4 Cambridge/London, UK http://www.mrc-bsu.cam.ac.uk/bugs/); 2003–7 [updated 2003–7; cited]; Available from: http://www.mrc-bsu.cam.ac.uk/bugs/

[B25] Caldwell DM Ades AE Higgins JP Simultaneous comparison of multiple treatments: combining direct and indirect evidence BMJ 2005 331 7521 897 900 10.1136/bmj.331.7521.897 16223826 PMC1255806

[B26] Sutton A Ades AE Cooper N Abrams K Use of indirect and mixed treatment comparisons for technology assessment Pharmacoeconomics 2008 26 9 753 67 10.2165/00019053-200826090-00006 18767896

[B27] Lu G Ades AE Combination of direct and indirect evidence in mixed treatment comparisons Stat Med 2004 23 20 3105 24 10.1002/sim.1875 15449338

[B28] Bucher HC Guyatt GH Griffith LE Walter SD The results of direct and indirect treatment comparisons in meta-analysis of randomized controlled trials J Clin Epidemiol 1997 50 6 683 91 10.1016/S0895-4356(97)00049-8 9250266

[B29] Glenny AM Altman DG Song F Sakarovitch C Deeks JJ D'Amico R Indirect comparisons of competing interventions Health Technol Assess 2005 9 26 1 134 16014203 10.3310/hta9260

[B30] Ades AE Sculpher M Sutton A Abrams K Cooper N Welton N Bayesian methods for evidence synthesis in cost-effectiveness analysis Pharmacoeconomics 2006 24 1 1 19 10.2165/00019053-200624010-00001 16445299

[B31] Office for National Statistics United Kingdom Interim Life Tables 1980–82 to 2008–10 2011 http://www.ons.gov.uk/ons/rel/lifetables/interim-life-tables/2008-2010/rft-ilt-uk-2008-2010.xls (Accessed: 03 December 2012)

[B32] MIMS Haymarket Business Media 2012 London, UK

[B33] Curtis L Unit costs of health and social care Canterbury: Personal and Social Services Research Unit 2011 Available at: http://www.pssru.ac.uk/pdf/uc/uc2011/uc2011.pdf. [Accessed 12 November 2012]

[B34] McHugh N Chandler D Griffiths C Helliwell P Lewis J McInnes I BSR guideline for anti-TNFa therapy in psoriatic arthritis 2004 London: British Society for Rheumatology 10.1093/rheumatology/keh514 15695305

[B35] The Monthly Index of Medical Specialities (MIMS) http://www.mims.co.uk [Accessed: 12 November 2012]

[B36] Kobelt G Jönsson L Lindgren P Young AKE Modelling the progression of rheumatoid arthritis:a two-country model to estimate costs to estimate costs and consequences of rheumatoid arthritis Arthritis Rheum 2002 46 2310 2319 10.1002/art.10471 12355478

[B37] Department of Health Reference costs 2007–2008 London: NHS

[B38] Hartman M Prins M Swinkels OQ Cost-effectiveness analysis of a psoriasis care instruction programme with dithranol compared with UVB phototherapy and inpatient dithranol treatment; Brit J Dermatol 2003 147 538 44 10.1046/j.1365-2133.2002.04920.x 12207597

[B39] Mease PJ Goffe BS Metz J VanderStoep A Finck B Burge DJ Etanercept in the treatment of psoriatic arthritis and psoriasis: a randomised trial Lancet 2000 356 385 390 10.1016/S0140-6736(00)02530-7 10972371

[B40] Mease PJ Kivitz AJ Burch FX Siegel EL Cohen SB Ory P Etanercept treatment of psoriatic arthritis: safety, efficacy, and effect on disease progression Arthritis Rheum 2004 50 7 2264 2272 July 2004 10.1002/art.20335 15248226

[B41] Sterry W Ortonne JP Kirkham B Brocq O Robertson D Pedersen RD Comparison of two etanercept regimens for treatment of psoriasis and psoriatic arthritis: PRESTA randomised double blind multicentre trial BMJ 2010 340 c147 10.1136/bmj.c147 20124563

[B42] Atzeni F Boccassini L Antivalle M Salaffi F Sarzi-Puttini P Etanercept plus ciclosporin versus etanercept plus methotrexate for maintaining clinical control over psoriatic arthritis: a randomised pilot study Ann Rheum Dis 2011 70 4 712 4 10.1136/ard.2010.130864 20810394

[B43] Antoni C Krueger GG de Vlam K Birbara C Beutler A Guzzo C Infliximab improves signs and symptoms of psoriatic arthritis: results of the IMPACT 2 trial Ann Rheum Dis 2005 64 8 1150 7 10.1136/ard.2004.032268 15677701 PMC1755609

[B44] Antoni CE Kavanaugh A Kirkham B Tutuncu Z Burmester GR Schneider U Sustained Benefits of Infliximab Therapy for Dermatologic and Articular Manifestations of Psoriatic Arthritis. Results From the Infliximab Multinational Psoriatic Arthritis Controlled Trial (IMPACT) Arthritis Rheum 2005 52 4 1227 1236 April 2005 10.1002/art.20967 15818699

[B45] Baranauskaite A Raffayova H Kungurov N Kubanova A Venalis A Helmle L Infliximab plus methotrexate is superior to methotrexate alone in the treatment of psoriatic arthritis in methotrexate-naive patients: the RESPOND study Ann Rheum Dis 2012 71 4 541 8 10.1136/ard.2011.152223 21994233 PMC3298666

[B46] Torii H Nakagawa H Japanese Infliximab Study i. Infliximab monotherapy in Japanese patients with moderate-to-severe plaque psoriasis and psoriatic arthritis. A randomized, double-blind, placebo-controlled multicenter trial J Dermatol Sci 2010 59 1 40 9 10.1016/j.jdermsci.2010.04.014 20547039

[B47] Mease PJ Gladman DD Ritchlin CT Ruderman EM Steinfeld SD Choy EH Adalimumab for the treatment of patients with moderately to severely active psoriatic arthritis: results of a double-blind, randomized, placebo-controlled trial Arthritis Rheum 2005 52 10 3279 89 10.1002/art.21306 16200601

[B48] Genovese MC Mease PJ Thomson GT Kivitz AJ Perdok RJ Weinberg MA Safety and efficacy of adalimumab in treatment of patients with psoriatic arthritis who had failed disease modifying antirheumatic drug therapy J Rheumatol 2007 34 5 1040 50 17444593

[B49] van Kuijk AWR Gerlag DM Vos K Wolbink G de Groot M de Rie MA A prospective, randomised, placebo-controlled study to identify biomarkers associated with active treatment in psoriatic arthritis: effects of adalimumab treatment on synovial tissue Ann Rheum Dis 2009 68 8 1303 9 10.1136/ard.2008.091389 18647851 PMC2703703

[B50] Cummins E Asseburg C Prasad M Buchanan J Punekar YS Cost effectiveness of golimumab for the treatment of active psoriatic arthritis Eur J Health Econ 2012 13 6 801 9 10.1007/s10198-011-0335-x 21720868

[B51] Pereda CA Nishishinya MB Martinez Lopez JA Carmona L Efficacy and safety of DMARDs in psoriatic arthritis: a systematic review Clin Exp Rheumatol 2012 30 2 282 9 22339882

[B52] Palominos PE Gaujoux-Viala C Fautrel B Dougados M Gossec L Clinical outcomes in psoriatic arthritis: A systematic literature review Arthritis Care Res (Hoboken) 2012 64 3 397 406 10.1002/acr.21552 22147535

[B53] Helliwell PS Fitzgerald O Mease PJ Development of composite measures for psoriatic arthritis: a report from the GRAPPA 2010 annual meeting J Rheumatol 2012 39 2 398 403 10.3899/jrheum.111233 22298265

[B54] Helliwell PS FitzGerald O Fransen J Gladman DD Kreuger GG Callis-Duffin K The development of candidate composite disease activity and responder indices for psoriatic arthritis (GRACE project) Ann Rheum Dis 2013 72 6 986 91 10.1136/annrheumdis-2012-201341 22798567

[B55] Olivieri I de Portu S Salvarani C Cauli A Lubrano E Spadaro A The psoriatic arthritis cost evaluation study: a cost-of-illness study on tumour necrosis factor inhibitors in psoriatic arthritis patients with inadequate response to conventional therapy Rheumatology 2008 47 11 1664 70 10.1093/rheumatology/ken320 18725374 PMC2569134

