# Supplementary material for: Systematic review, network meta-analysis and economic evaluation of biological therapy for the management of active psoriatic arthritis
Source: BMC Musculoskelet Disord. 2014 Jan 20;15:26. doi: 10.1186/1471-2474-15-26 (PMC3903562; doi:10.1186/1471-2474-15-26)
Supplement: Additional file 1: Table S4 — Selection criteria for studies included in the meta-analysis. [file 1471-2474-15-26-S1.docx]

Table 4: Selection criteria for studies included in the meta-analysis

| Inclusion criteria | Description |
| --- | --- |
| Population | Adult patients (aged ≥18 years), with active and progressive PsA which have responded inadequately to previous disease-modifying anti-rheumatic drugs (DMARDs) |
| Interventions | All biologic DMARDs licensed in the UK  For the meta-analysis, studies needed to include one arm at a licensed dose (used with or without palliative care such as NSAIDs and conventional DMARDs), i.e.   - Etanercept 2x25 mg/week - Infliximab 5 mg/kg/8 weeks - Adalimumab 40 mg/2 weeks - Golimumab 50 kg/4 weeks |
| Study design | Prospective randomised placebo-controlled trials (Phase II-IV) |
| Outcomes | Psoriatic Arthritis Response Criteria (PsARC)  Health Assessment Questionnaire (HAQ)  Psoriasis Area and Severity Index (PASI) |
