# Supplementary figures and images for: Systematic review, network meta-analysis and economic evaluation of biological therapy for the management of active psoriatic arthritis
Source: BMC Musculoskelet Disord. 2014 Jan 20;15:26. doi: 10.1186/1471-2474-15-26 (PMC3903562; doi:10.1186/1471-2474-15-26)

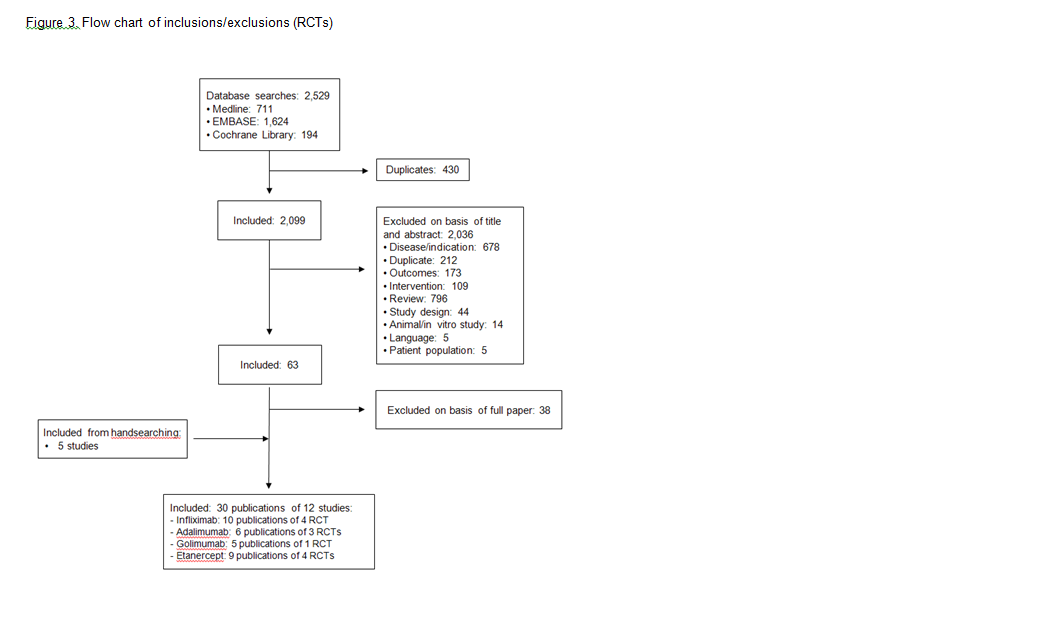

Supplement: Additional file 2: Figure S3 — Flow chart of inclusions/exclusions (RCTs). [file 1471-2474-15-26-S2.png]

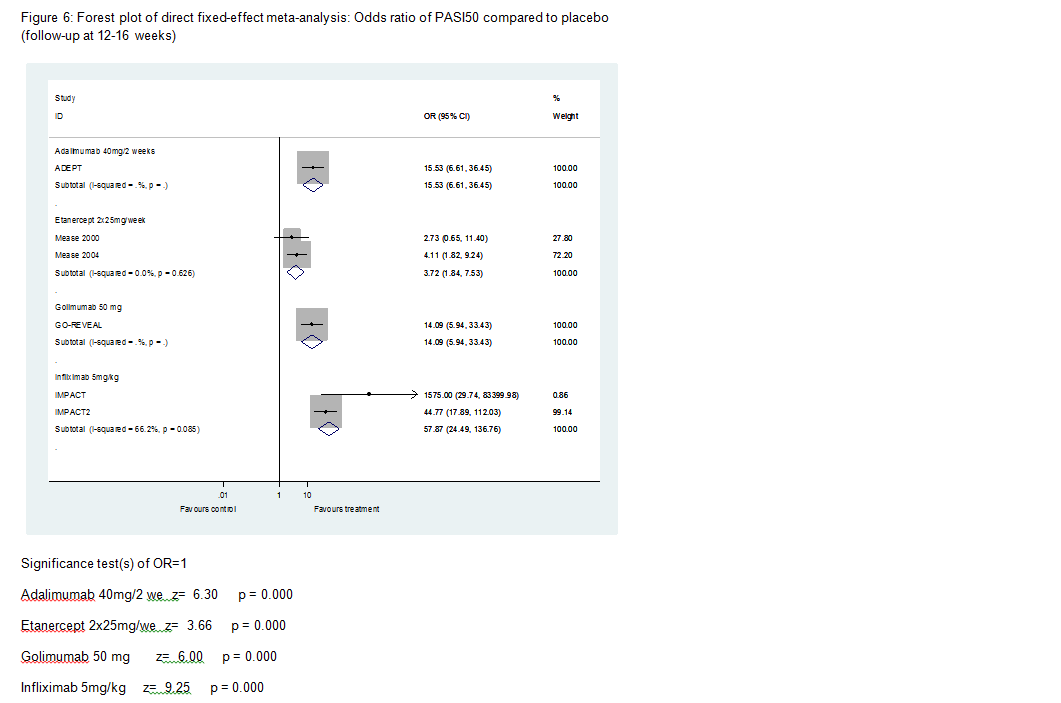

Supplement: Additional file 6: Figure S6 — Forest plot of direct fixed-effect meta-analysis: Odds ratio of PASI50 compared to placebo (follow-up at 12–16 weeks). [file 1471-2474-15-26-S6.png]

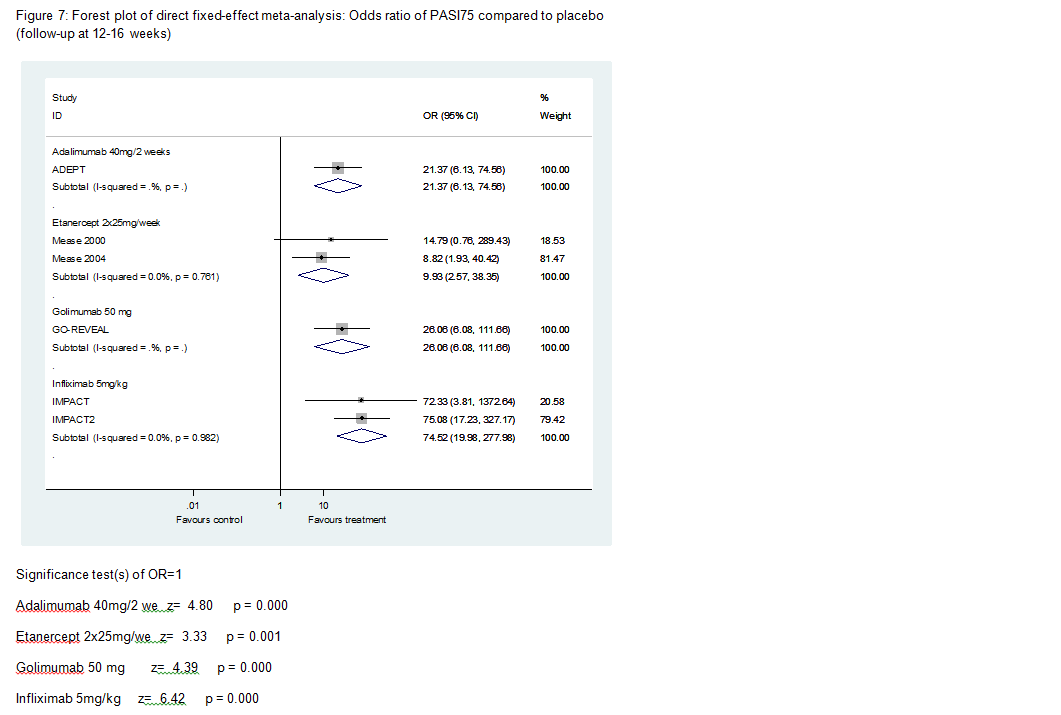

Supplement: Additional file 7: Figure S7 — Forest plot of direct fixed-effect meta-analysis: Odds ratio of PASI75 compared to placebo (follow-up at 12–16 weeks). [file 1471-2474-15-26-S7.png]

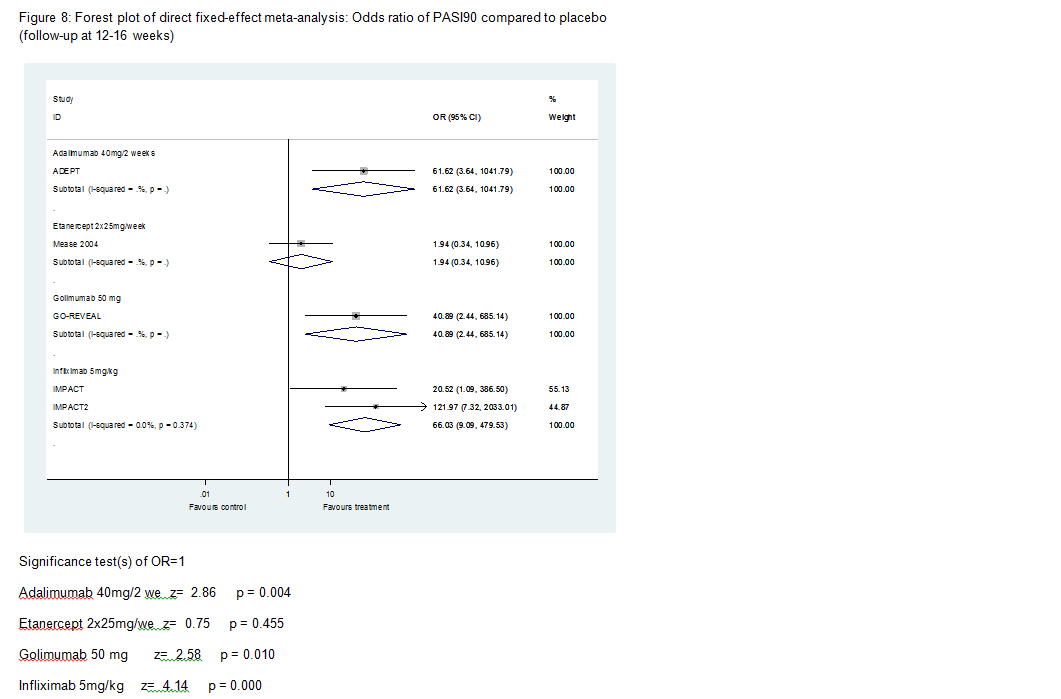

Supplement: Additional file 8: Figure S8 — Forest plot of direct fixed-effect meta-analysis: Odds ratio of PASI90 compared to placebo (follow-up at 12–16 weeks). [file 1471-2474-15-26-S8.png]

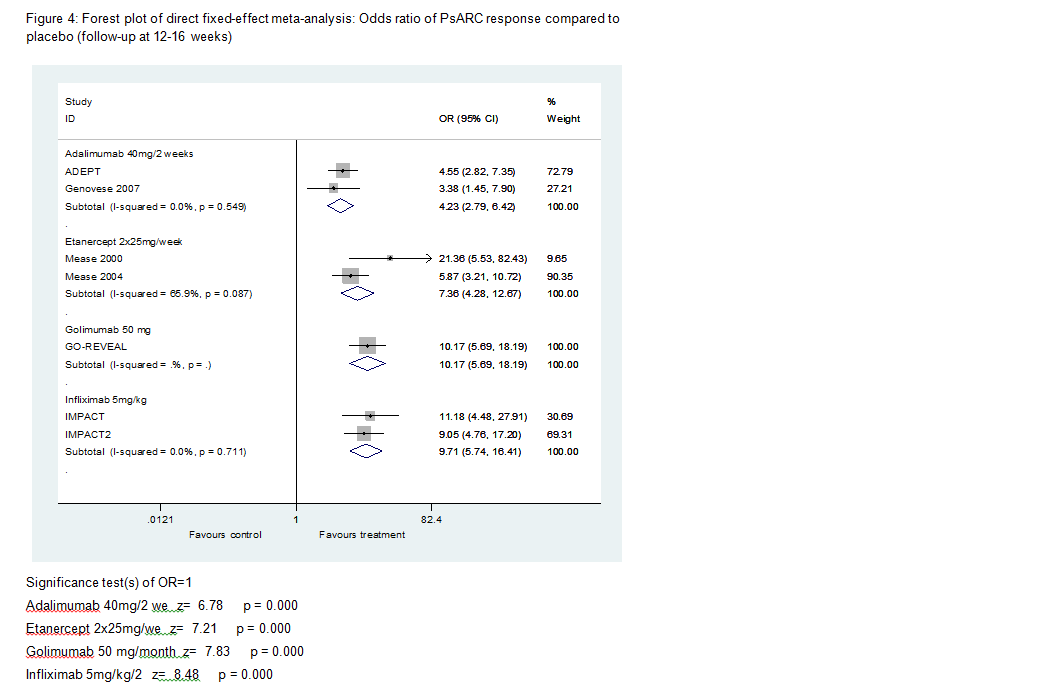

Supplement: Additional file 9: Figure S4 — Forest plot of direct fixed-effect meta-analysis: Odds ratio of PsARC response compared to placebo (follow-up at 12–16 weeks). [file 1471-2474-15-26-S9.png]

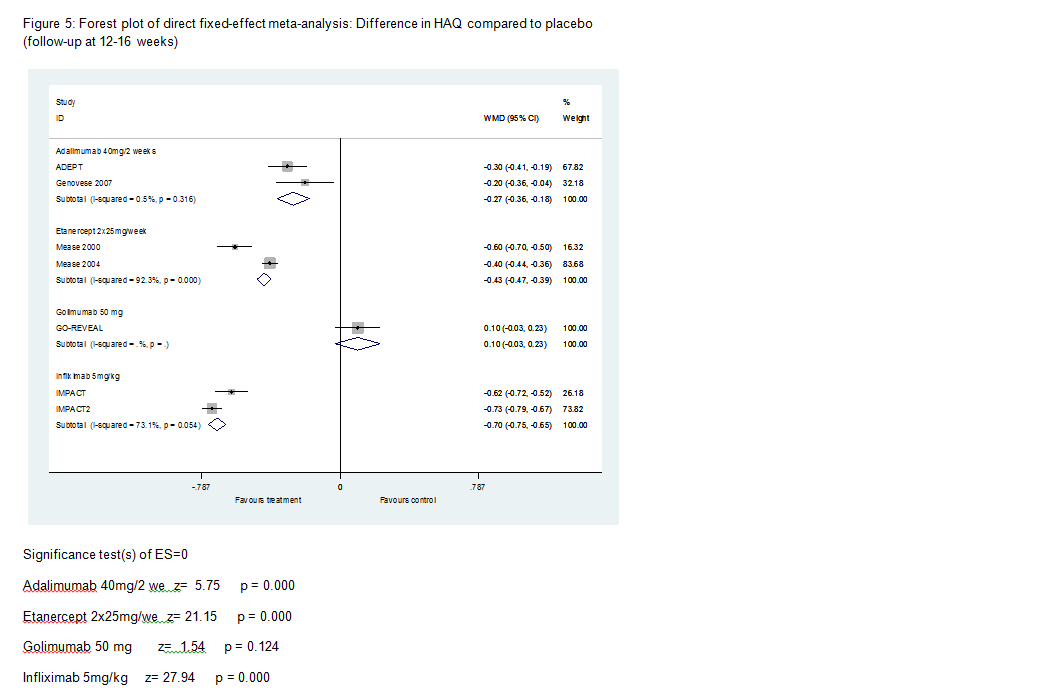

Supplement: Additional file 10: Figure S5 — Forest plot of direct fixed-effect meta-analysis: Difference in HAQ compared to placebo (follow-up at 12–16 weeks). [file 1471-2474-15-26-S10.png]

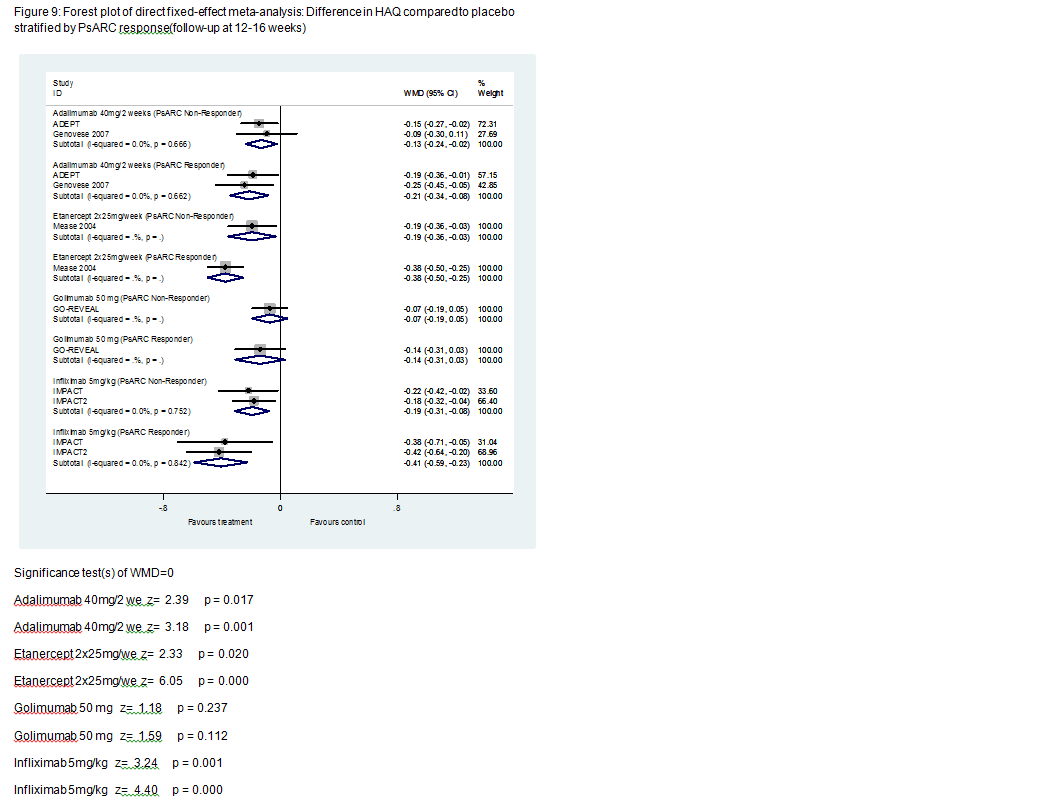

Supplement: Additional file 11: Figure S9 — Forest plot of direct fixed-effect meta-analysis: Difference in HAQ compared to placebo stratified by PsARC response (follow-up at 12–16 weeks). [file 1471-2474-15-26-S11.png]
