# Supplementary material for: Systematic review, network meta-analysis and economic evaluation of biological therapy for the management of active psoriatic arthritis
Source: BMC Musculoskelet Disord. 2014 Jan 20;15:26. doi: 10.1186/1471-2474-15-26 (PMC3903562; doi:10.1186/1471-2474-15-26)
Supplement: Additional file 3: Table S5 — Summary of studies included in the meta-analysis. [file 1471-2474-15-26-S3.docx]

Table 5: Summary of studies included in the meta-analysis

| Study | Treatment arm | Age, years; mean (SD)  [range] | Male, n (%) | Duration of PsA, years; mean (SD) | Number of prior DMARDs; mean (SD) | Concomitant therapy | No. of patients | Duration of follow up |
| --- | --- | --- | --- | --- | --- | --- | --- | --- |
| Adalimumab (n=2) | | | | | | | | |
| ADEPT ([38](#_ENREF_38))  Full paper  Double-blind RCT  USA, Canada, UK | ADA, 40 mg every other week^¶^ [+ MTX in 51% of patients] | 48.6 (12.5) | (56.3) | 9.8 (8.3) | 1.5 (1.2) | Steroids or MTX >30 mg/week not permitted | 153 | 24 weeks^††^ |
|  | Placebo [+ MTX in 50% of patients] | 49.2 (11.1) | (54.9) | 9.2 (8.7) | 1.5 (1.2) |  | 162 |  |
| Genovese 2007 ([39](#_ENREF_39))  Full paper  Double-blind RCT | ADA, 40 mg every other week [+ MTX^¶¶^ in 64.7% of patients] | 50.4 (11.0) | 29 (56.9) | 7.5 (7.0) | 1.7 (0.9) | CS^§§^: 7.8%  NSAIDs^§§^: 72.6% | 51 | 12 weeks^†††^ |
|  | Placebo [+ MTX^¶¶^ in 67.3% of patients] | 47.7 (11.3) | 25 (51.0) | 7.2 (7.0) | 2.1 (1.3) | CS^§§^: 18.4%  NSAIDs^§§^: 85.7% | 49 |  |
| Etanercept (n=2) | | | | | | | | |
| Mease 2000 ([30](#_ENREF_30))  Full paper  Double-blind RCT  USA | ETN, 25 mg twice weekly | 46.0  [30.0–70.0] | 16 (53) | 9.0  (1.0–31.0) | 1.5  (0–4) | CS: 20%  NSAIDs: 67%  MTX: 47% | 30 | 12 weeks |
|  | Placebo | 43.5  [24.0–63.0] | 18 (60) | 9.5  (1.0–30.0) | 2.0  (1.0–5.0) | CS: 40%  NSAIDs: 77%  MTX: 47% [mean MTX 16.3 mg/week] | 30 |  |
| Mease, 2004 ([31](#_ENREF_31))  Full paper  Double-blind RCT  USA | ETN, 25 mg twice weekly | 47.6 | 58 (57) | 9.0 | 1.7 | CS: 19%  NSAIDs: 88%  MTX: 42% [mean MTX 16.3 mg/week] | 101 | 24 weeks  (48 week extension^§^) |
|  | Placebo | 47.3 | 47 (45) | 9.2 | 1.6 | CS: 15%  NSAIDs: 83%  MTX: 41%  [mean MTX 15.4 mg/week] | 104 |  |
| Golimumab (n=1) | | | | | | | | |
| GO-REVEAL ([10](#_ENREF_10))  Full paper  Double-blind RCT  USA, UK, Canada, Spain | GOL, 50 mg every 4 weeks^†^ | 45.7 (10.7) | 89 (61) | 7.2 (6.8) | All patients treated with cDMARD; bDMARD naive | CS: 13%  NSAIDs: 75%  MTX: 49% [mean MTX 14.8 mg/week] | 146 | Primary publication: 24 weeks  [Additional abstracts([50-52](#_ENREF_50)) up to 104 weeks^‡^] |
|  | GOL, 100 mg every 4 weeks^†^ | 48.2 (10.9) | 86 (59) | 7.7 (7.8) |  | CS: 18%  NSAIDs: 75%  MTX: 47% [mean MTX 15.5 mg/week] | 146 |  |
|  | Placebo^†^ | 47.0 (10.6) | 69 (61) | 7.6 (7.9) |  | CS: 17%  NSAIDs: 78%  MTX: 48% [mean MTX 15.0 mg/week] | 113 |  |
| Infliximab (n=2) | | | | | | | | |
| IMPACT ([35](#_ENREF_35))  Full paper  Double-blind RCT  Europe, USA, Canada | IFX, 5 mg/kg at weeks 0, 2, 6 and 14 (Phase I)^†^ | 45.7 (11.1) | 30 (57.7) | 11.7 (9.8) | ≥1 DMARD | CS, NSAIDs and cDMARD [33/52, 63%] permitted^‡^ | 52 | 98 weeks (double blind up to 16 weeks) |
|  | Placebo (Phase I)^†^ | 45.2 (9.7) | 30 (57.7) | 11.0 (6.6) |  | CS, NSAIDs and cDMARD [41/52, 79%] permitted^§^ | 52 |  |
| IMPACT2 ([34](#_ENREF_34))  Full paper  Double-blind RCT  Europe, USA, Canada | IFX, 5 mg/kg at weeks 0, 2, 6, 14 and 22^††^,^§§^ | 47.1 (12.8) | (71) | 8.4 (7.2) | ≥1 DMARD [TNF-α inhibitor naïve] | CS: 15%  NSAIDs: 71%  MTX^¶^: 47% | 100 | 54 weeks (early escape at week 16^‡‡^) |
|  | Placebo^††^, ^‡‡^ | 46.5 (11.3) | (51) | 7.5 (7.8) |  | CS: 10%  NSAIDs: 73%  MTX^¶^: 45% | 100 |  |

ADA, adalimumab; bDMARD, biological disease modifying anti-rheumatic drug; cDMARD, conventional disease modifying anti-rheumatic drug; CS, corticosteroids; GOL, golimumab; ETN, etanercept; MTX, methotrexate; NR, not reported; NSAIDs, non-steroidal anti-inflammatory drugs; PsA, psoriatic arthritis

^¶^After week 12, patients who failed to have ≥20% decrease in both swollen and tender joint count on two consecutive visits could receive rescue therapy with corticosteroids or DMARDs

^¶¶^Maximum dose of 30 mg/week

^††^All patients who completed the 24-week protocol were eligible for long-term treatment with 40 mg ADA eow in an open-label extension for up to 120 weeks. After 12 weeks in the extension study, patients who did not have a ≥20% improvement compared with baseline in the tender joint count and the swollen joint count were allowed to increase the ADA dose to 40 mg/week (n=54, 18.9%)

^†††^Patients who completed the blinded phase could elect to receive open-label ADA 40 mg eow

^§^Patients who completed the 24-week randomised phase were given the option to continue maintenance therapy, according to their randomised treatment until all patients had completed the randomised portion of the study. The final period comprised open-label treatment of all eligible patients with ETN in a 48-week extension.

^†^At week 16, patients with <10% improvement from baseline in both the swollen and tender joint counts entered early escape, with dose escalation from placebo to golimumab 50 mg or from golimumab 50 mg to golimumab 100 mg. Patients in the golimumab 100 mg group meeting early escape criteria continued with the 100-mg dose in a blinded manner. Beginning at week 24, all patients received golimumab and continued to receive subcutaneous treatment every 4 weeks. The last evaluation for this study was performed at week 24.

^‡^All patients randomised to placebo receive GOL 50 mg from week 24 through week 104. Patients on GOL 50 mg could be dose escalated based on the investigator’s assessment to GOL 100 mg after unblinding at week 52.

^¶^After week 12, patients who failed to have ≥20% decrease in both swollen and tender joint count on two consecutive visits could receive rescue therapy with corticosteroids or DMARDs

^††^All patients who completed the 24-week protocol were eligible for long-term treatment with 40 mg ADA eow in an open-label extension for up to 120 weeks. After 12 weeks in the extension study, patients who did not have a ≥20% improvement compared with baseline in the tender joint count and the swollen joint count were allowed to increase the ADA dose to 40 mg/week (n=54, 18.9%)

^§§^Use at baseline
