# Supplementary material for: Systematic review, network meta-analysis and economic evaluation of biological therapy for the management of active psoriatic arthritis
Source: BMC Musculoskelet Disord. 2014 Jan 20;15:26. doi: 10.1186/1471-2474-15-26 (PMC3903562; doi:10.1186/1471-2474-15-26)
Supplement: Additional file 4: Table S6 — Data used in NMA: PsARC response, change in HAQ compared to baseline and PASI 50/75/90. [file 1471-2474-15-26-S4.docx]

Table 6: Data used in NMA: PsARC response, change in HAQ compared to baseline and PASI 50/75/90

| Study | Treatment arm | Follow up (weeks) | No. of patients randomised | PsARC, n | Ref | Mean change in HAQ | SE in change in HAQ | Ref | No. in PASI subset | PASI 50/75/90, n | Ref |
| --- | --- | --- | --- | --- | --- | --- | --- | --- | --- | --- | --- |
| ADEPT | Adalimumab 40mg/2 weeks | 12 | 153 | 94 | Mease 2005 ([38](#_ENREF_38)) | -0.40 | 0.041 | Mease 2005 ([38](#_ENREF_38)) | 69 | 50/34/21 | Mease 2005 ([38](#_ENREF_38)) |
|  | Placebo | 12 | 162 | 42 |  | -0.10 | 0.039 |  | 69 | 10/3/0 |  |
| Genovese 2007 | Adalimumab 40mg/2 weeks | 12 | 51 | 26 | Genovese 2007 ([39](#_ENREF_39)) | -0.30 | 0.070 | Genovese 2007 ([39](#_ENREF_39)) | - | -/-/- | No data |
|  | Placebo | 12 | 51 | 12 |  | -0.10 | 0.043 |  | - | -/-/- |  |
| Mease 2000 | Etanercept 2x25mg/week | 12 | 30 | 26 | Mease 2000 ([30](#_ENREF_30)) | -0.70 | 0.050 | CSR 016.0612  (unpublished) ([53](#_ENREF_53)) | 19 | 8/5/- | Mease 2000 ([30](#_ENREF_30)); Rodgers 2011^†^ ([12](#_ENREF_12)) |
|  | Placebo | 12 | 30 | 7 |  | -0.10 | 0.008 |  | 19 | 4/0/- |  |
| Mease 2004 | Etanercept 2x25mg/week | 12 | 101 | 73 | Mease 2004 ([31](#_ENREF_31)) | -0.50 | 0.022 | CSR 016.0030 (unpublished) ([54](#_ENREF_54)); Rodgers 2011^†^ ([12](#_ENREF_12)) | 66 | 31/15/4 | Mease 2004 ([31](#_ENREF_31)) Rodgers 2011^†^ ([12](#_ENREF_12)) |
|  | Placebo | 12 | 104 | 32 |  | -0.10 | 0.004 |  | 62 | 11/2/2 |  |
| GO-REVEAL | Golimumab 50 mg | 14 | 146 | 107 | Kavanaugh 2009 ([10](#_ENREF_10)) | -0.30 | 0.046 | Golimumab STA  Report 2010^†^ ([55](#_ENREF_55)) | 109 | 63/44/22 | Kavanaugh 2009 ([10](#_ENREF_10)) |
|  | Golimumab 100 mg | 14 | 146 | 105 |  | - | - |  | 108 | 83/63/26 |  |
|  | Placebo | 14 | 113 | 24 |  | -0.40 | 0.046 |  | 79 | 7/2/0 |  |
| IMPACT | Infliximab 5mg/kg | 16 | 52 | 39 | Antoni 2005a ([35](#_ENREF_35)) | -0.60 | 0.049 | Antoni 2005a ([35](#_ENREF_35)) | 22 | 22/15/8 | Antoni 2005a ([35](#_ENREF_35)) |
|  | Placebo | 16 | 52 | 11 |  | 0.02 | 0.002 |  | 17 | 0/0/0 |  |
| IMPACT2 | Infliximab 5mg/kg | 14 | 100 | 77 | Antoni 2005b ([34](#_ENREF_34)) | -0.53 | 0.023 | Antoni 2005b ([34](#_ENREF_34)) | 83 | 68/53/34 | Antoni 2005b ([34](#_ENREF_34)) |
|  | Placebo | 14 | 100 | 27 |  | 0.20 | 0.018 |  | 87 | 8/2/0 |  |

PsARC, Psoriatic Arthritis Response Criteria, HAQ, health assessment questionnaire; SE, standard error ^†^Data from secondary published source
