# Supplementary material for: Systematic review, network meta-analysis and economic evaluation of biological therapy for the management of active psoriatic arthritis
Source: BMC Musculoskelet Disord. 2014 Jan 20;15:26. doi: 10.1186/1471-2474-15-26 (PMC3903562; doi:10.1186/1471-2474-15-26)
Supplement: Additional file 5: Table S7 — Data used in NMA: Conditional Data for change in HAQ | PsARC response compared to baseline. [file 1471-2474-15-26-S5.docx]

Table 7: Data used in NMA: Conditional Data for change in HAQ | PsARC response compared to baseline

| Study | Data source | Treatment arm | PsARC Responders | | PsARC Non-responders | |
| --- | --- | --- | --- | --- | --- | --- |
|  |  |  | Mean change in HAQ | SE in Change in HAQ | Mean change in HAQ | SE in Change in HAQ |
| ADEPT | Rodgers 2011^†^ ([12](#_ENREF_12)) | Placebo | -0.313 | 0.076 | 0.026 | 0.037 |
|  |  | Adalimumab 40mg/2 weeks | -0.500 | 0.045 | -0.120 | 0.053 |
| Genovese 2007 | Rodgers 2011^†^ ([12](#_ENREF_12)) | Placebo | -0.177 | 0.062 | -0.057 | 0.053 |
|  |  | Adalimumab 40mg/2 weeks | -0.423 | 0.081 | -0.150 | 0.090 |
| Mease 2000 | No data | Placebo | - | - | - | - |
|  |  | Etanercept 2x25mg/week | - | - | - | - |
| Mease 2004 | Rodgers 2011^†^ ([12](#_ENREF_12)) | Placebo | –0.258 | 0.006 | –0.002 | 0.042 |
|  |  | Etanercept 2x25mg/week | -0.635 | 0.062 | -0.196 | 0.072 |
| GO-REVEAL | Golimumab STA^‡^;  Cummins 2011^†^ ([41](#_ENREF_41)) | Placebo | -0.286 | 0.050 | 0.023 | 0.024 |
|  |  | Golimumab 50 mg | -0.424 | 0.071 | -0.049 | 0.056 |
| IMPACT | Rodgers 2011^†^ ([12](#_ENREF_12)) | Placebo | -0.270 | 0.140 | 0.020 | 0.050 |
|  |  | Infliximab 5mg/kg | -0.650 | 0.090 | -0.200 | 0.090 |
| IMPACT2 | Rodgers 2011^†^ ([12](#_ENREF_12)) | Placebo | -0.160 | 0.096 | 0.070 | 0.042 |
|  |  | Infliximab 5mg/kg | -0.580 | 0.057 | -0.110 | 0.060 |

CSR, clinical study report; HAQ, health assessment questionnaire; PsARC, Psoriatic Arthritis Response Criteria; SE, standard error

^†^Data from secondary published source; ^‡^data censored in this report
